# LncRNA MONC suppresses the malignant phenotype of Endometrial Cancer Stem Cells and Endometrial Carcinoma Cells by regulating the MiR-636/GLCE axis

**DOI:** 10.1186/s12935-021-01911-1

**Published:** 2021-06-30

**Authors:** Yibing Li, Jianing Huo, Junjian He, Xiaoxin Ma

**Affiliations:** Department of Obstetrics and Gynecology, Shengjing Hospital of China Medical University, No 39 Huaxiang Road, Tiexi District, Shenyang, 110000 Liaoning People’s Republic of China

**Keywords:** Endometrial carcinoma, Cancer stem cells, LncRNA MONC, MicroRNA-636, GLCE

## Abstract

**Background:**

Emerging evidence shows that abnormal expression of long non-coding RNA is involved in the occurrence and development of various tumors. LncRNA MONC is abnormally expressed in head and neck squamous cell carcinoma, lung cancer, colorectal cancer, and acute megakaryocytic leukemia, but the biological function and potential regulatory mechanism of MONC in endometrial cancer stem cells (ECSCs) and endometrial cancer cells (ECCs) have not been studied. In this study, we aimed to explore the tumor suppressive effect and mechanism of MONC in regulating ECSCs and ECCs.

**Methods:**

We used qRT-PCR to detect the expression of MONC, miR-636 and GLCE in normal human endometrial tissues and endometrial carcinoma (EC) tissues. Luciferase assay was used to verify the binding sites between MONC and miR-636 and between miR-636 and GLCE. Double fluorescence in situ hybridization was used to locate MONC and miR-636 in cells. ECSCs were obtained by flow cytometry sorting assay. Sphere formation assay, CCK-8 assay, transwell invasion assay, cell cycle analysis and apoptosis assay were used to detect the effects of MONC/miR-636/GLCE axis on the malignant biological behavior of ECSCs and ECCs. The effect of MONC on the epithelial-to-mesenchymal transition (EMT) process was detected using western blot. Finally, we conducted in vivo verification through Tumor xenografts in BALB/C nude mice.

**Results:**

In this study, we found MONC is low expression in endometrial carcinoma (EC) and patients in the MONC high-expression group had a better prognosis. MONC and miR-636 are relatively co-localized in the cytoplasm. MONC directly inhibits the malignant biological behavior of ECSCs and ECCs by directly inhibiting miR-636. Simultaneously, miR-636 may indirectly reduce the expression of MONC. Down-regulation of miR-636 may promote GLCE expression by targeting the 3′-untranslated region (UTR) of the downstream gene GLCE, thereby inhibiting the progression of ECSCs. MONC combined with miR-636 inhibited tumor epithelial-to-mesenchymal transition (EMT) process. In addition, we verified the tumor suppressive effect of MONC in nude mice, miR-636 can rescue the tumor suppressive effect of overexpressing MONC.

**Conclusions:**

In conclusion, this study showed that MONC inhibits the malignant phenotypes of ECSCs and ECCs by regulating the miR-636/GLCE axis. Thus the MONC/miR-636/GLCE axis may provide novel treatment avenues for human EC.

**Supplementary Information:**

The online version contains supplementary material available at 10.1186/s12935-021-01911-1.

## Background

Endometrial carcinoma (EC) is one of the three major malignant tumors in gynecology, with 61,880 new cases each year in the United States, second only to breast cancer, lung cancer, bronchial cancer, and colorectal cancer. There are 12,160 deaths each year due to EC, second only to lung cancer, bronchial cancer, breast cancer, colorectal cancer, pancreatic cancer, and ovarian cancer [1]. Risk factors for EC include elevated estrogen levels (caused by obesity, diabetes, and high-fat diets), premature menarche, non-parturients, late desperate age, Lynch syndrome, grade ≥ 55 years old, and tamoxifen use [2].

Cancer stem cells (CSCs) are a class of cells that have the unique characteristics of self-renewal and the ability to differentiate into heterogeneous lineages of cancer cells [3, 4]. CSCs can initiate tumor formation and promote tumor cell proliferation, while differentiation of component tumor cells plays a vital role in the occurrence, development, metastasis, recurrence, and drug resistance of malignant tumors [5, 6]. In our previous research, we used serum-free suspension culture to isolate endometrial cancer stem cells (ECSCs) from Ishikawa cells [7].

Long non-coding RNA (LncRNA) is a non-coding RNA with a length of more than 200 nucleotides. In recent years, LncRNA has been found to be an important biological marker for the diagnosis of tumors and their [8]. Increasing number of studies have shown that LncRNA plays a vital role in the occurrence and development of tumors, including EC. LncRNA is dysregulated in EC and is closely related to tumorigenesis, metastasis, and chemoresistance [9]. However, LncRNA has been rarely studied in ECSCs. LncRNA MONC, mir-99a-let-7c cluster host gene, also known as MIR99AHG, is a good prognostic indicator of head and neck squamous cell carcinoma [10], lung squamous cell carcinoma [11], and colorectal cancer [12]. In acute megakaryoblastic leukemia (AML), MONC acts as an oncogene to promote leukemia growth in AML cell lines and primary patient samples [13]. However, MONC has not been studied in EC and ECSCs.

MicroRNA (miRNA) is a non-coding RNA approximately 20–24 nucleotides in length, that can induce translational inhibition or degradation of target mRNA to inhibit gene expression [14]. Abnormally expressed miRNAs are associated with tumorigenesis, development, and response to treatment [15]. In recent years, miR-636 has been studied in tumors and was fond to be abnormally expressed in bladder cancer and liver cancer [16, 17]. However, miR-636 has not been studied in EC and ECSCs.

In this study, we aimed to study the expression level of MONC and its interaction with miR-636 in EC tissues and ECSCs. It was found that miR-636 targeted MONC in a sequence-specific manner in the cytoplasm, suggesting that there may be mutual inhibition between miR-636 and MONC. To study the potential mechanism in this process, the effect of MONC on miR-636-induced GLCE regulation and its effect on epithelial-to-mesenchymal transition (EMT) were also studied. Our findings shed light on new molecular mechanisms for the progression of EC and provide potential treatment options.

## Materials and methods

### Human tissue specimens

All EC tissue samples and normal endometrial tissue samples were obtained from patients undergoing total hysterectomy at Shengjing Hospital of China Medical University. The weight of each tissue sample is 500 mg. The pathological type of the 60 cases of EC tissue samples we collected were endometrial adenocarcinoma. The diagnosis of EC was evaluated by two experienced clinical pathologists based on FIGO for histological diagnosis. No patients received chemotherapy, radiotherapy, hormones, or treatment before surgery. According to the WHO standard, the endometrial adenocarcinoma is divided into high differentiation, middle differentiation and low differentiation. Informed consent was obtained from all patients and the study was approved by the Ethics Committee of Shengjing Hospital, China Medical University (2018PS251K).

### ECSCs, cell lines, and cell culture

ECSCs were cultured in serum-free medium, DMEM/F12 (1:1) (Corning, New York, USA) containing 2% B27 Supplement (Gibco, New York, USA), 20 ng/mL EGF (PeproTech, New Jersey, USA), 20 ng/mL bFGF (PeproTech), and 1% penicillin–streptomycin (Invitrogen, Carlsbad, California, USA). Ishikawa cell line (Shanghai huiying, Shanghai, China) and HEC-1A cell line (Genechem, Shanghai, China) were cultured in α-MEM medium (Bioind, Kibbutz Beit Haemek, Israel) and McCoy's 5A medium (Bioind), respectively. The medium contained 10% fetal bovine serum (FBS) (Bioind) and 1% penicillin–streptomycin (Invitrogen). HEK293T cells were cultured in DMEM/high-glucose medium (Corning). The medium contained 10% FBS (Bioind) and 1% penicillin–streptomycin (Invitrogen). All cells were cultured in a humidified incubator at 37° C with 5% CO_2_.

### RNA extraction, reverse transcription, and quantitative real-time polymerase chain reaction (qRT-PCR)

Total RNA was extracted from tissues and cells using TRIzol reagent (Takara, Beijing, China). The complementary DNAs (cDNAs) for the LncRNAs and mRNAs of interest were reverse-transcribed from 2 μg total RNA using PrimeScript RT-polymerase (Takara). The cDNAs for the miRNAs of interest were synthesized from 1 μg total RNA using miRNA 1st Strand cDNA Synthesis SuperMix (Vazyme, Nanjing, China). We performed qRT-PCR using SYBR-Green Premix (Takara), miRNA Universal SYBR® qPCR Master Mix (Vazyme), and specific PCR primers (Sangon Biotech, Shanghai, China). Glyceraldehyde-3-phosphate dehydrogenase (GAPDH) and U6 were used as internal controls. We determined the expression of LncRNA, mRNA, and miRNA using the calculating 2^−ΔΔCT^ method. Primer sequences are summarized in Table 1.

**Table 1:** Primer Sequences

Gene name	Primer Sequence
MONC	Forward: CCAGACTTGTCGCACGGAT
	Reverse: AATGCACAGCAATCAGTTCCTC
miR-636	Forward: TGTGCTTGCTCGTCCCG
	Reverse: AGTGCAGGGTCCGAGGTATT
	RT Primer: GTCGTATCCAGTGCAGGGTCCGAGGTATTCGCACTGGATACGACTGCGGG
GLCE	Forward: TGAACCACGTGGCCAAACAA
	Reverse: TTCGTTCCCCTCTCGTCTCC
GAPDH	Forward: GCACCGTCAAGGCTGAGAAC
	Reverse: TGGTGAAGACGCCAGTGGA
U6	Forward: AGAGAAGATTAGCATGGCCCCTG
	Reverse: ATCCAGTGCAGGGTCCGAGG
	RT Primer: GTCGTATCCAGTGCAGGGTCCGAGGTATTCGCACTGGATACGACAAAATA

### Western blot

Protein was extracted from tissues and cells using RIPA Lysis Buffer (Beyotime Biotechnology, Shanghai, China) and phenylmethanesulfonyl fluoride (PMSF) (Beyotime Biotechnology). Protein denaturation was performed after adding SDS-PAGE Sample Loading Buffer. Denatured proteins were separated by sodium dodecyl sulfate polyacrylamide gel electrophoresis (SDS-PAGE) and transferred to polyvinylidene fluoride membranes (Millipore, USA). Membranes were blocked with 5% skim milk for 2 h and washed thrice with TBST. Membranes were then incubated with diluted primary antibodies against GLCE (Abcom, Cambridge, United Kingdom), Snail1 (Proteintech), E-cadherin (Proteintech), N-cadherin (Proteintech), and Vimentin (Proteintech), PCNA (Proteintech) overnight at 4 °C, and washed thrice with TBST thereafter. Membranes were then incubated with corresponding secondary antibodies for 2 h, followed by three washes with TBST. The protein bands were visualized using BeyoECL Star (Beyotime Biotechnology) and Quantum One imaging software (Bio-Rad, California, USA), and normalized to the gray intensity of GAPDH or β-actin.

### Transfection and generation of stably transfected cell line

MONC lentiviral overexpression and lentiviral knockdown plasmids were purchased from GeneChem (Shanghai, China). ECSCs and Ishikawa cells were then transfected at a multiplicity of infection (MOI) of 20, while an MOI of 10 was used for HEC-1A cells. MiR-636 agomir and antagomir were purchased from GenePharma (Shanghai, China), and GLCE overexpression and knockdown plasmids were purchased from GeneChem. All cells were transfected using jetPRIME® in vitro DNA and siRNA Transfection Reagent (PolyPlus-transfection, France). The relevant sequences are provided in Table 2. The lentiviral vector [LV-MONC-RNAi (67,379-1)] and plasmid [GLCE-RNAi(5952-1)] with the best knockdown effect were selected for subsequent experiments (Additional file 1: Figure S1A, S1B).

**Table 2:** Sequences of lentivirus, agomir/antagomir, and plasmid

Name	Sequence
LV-MONC-RNAi (67,379–1)	5′-GAGCGCAATTATTCCTCTAAA-3’
LV-MONC-RNAi (67,380–1)	5′-AAACTTAATGGAGGAGGCTGA-3’
LV-MONC-RNAi (67,381–1)	5′-GACAAGAGCACCTCAAAGGCA-3’
LV-CON077 (LV-MONC-RNAi)	5′-TTCTCCGAACGTGTCACGT-3’
AgomiR-636	Sense: 5′-UGUGCUUGCUCGUCCCGCCCGCA-3’
	Antisense: 5′-CGGGCGGGACGAGCAAGCACAUU-3’
Negative control	Sense: 5′-UUCUCCGAACGUGUCACGUTT-3’
	Antisense: 5′-ACGUGACACGUUCGGAGAATT-3’
AntagomiR-636	5′-UGCGGGCGGGACGAGCAAGCACA-3’
Inhibitor NC	5′-CAGUACUUUUGUGUAGUACAA-3'
GLCE-RNAi(5951–1)	5′-ccTCACATAGAGGTATATGAA-3’
GLCE-RNAi(5952–1)	5′-TGGCTGATAAGTCTAGATTCA-3’
GLCE-RNAi(5953–1)	5′-GTGTGCCATTATCTACACAAT-3’
CON036(GLCE-RNAi)	5′-TTCTCCGAACGTGTCACGT-3’

### Flow cytometry sorting assay

Flow cytometry sorting assay was performed as described in our previous study [7]. Briefly, Ishikawa cells were cultured and passaged in serum-free medium, and sphere formation was observed. The cell suspension was centrifuged and the cell pellet was washed once with phosphate-buffered saline (PBS). Cells(1 × 10^7^) were resuspended in 100 µL PBS and labeled with APC Mouse Anti-Human CD133 (BD Biosciences, New Jersey, USA) and PerCP-Cy7M 5.5 Mouse Anti-Human CD44 (BD Biosciences). Following incubation for 10 min in the dark, cells were washed with PBS, and the supernatant was discarded after centrifugation. Thereafter, cells were resuspended in PBS, and flow sorted using the BD FACSAriaTM III Cell Sorter (BD Biosciences). ECCs positive for both CD44 and CD133 were obtained using flow cytometry cell sorting. We obtained ECSCs via flow sorting, and these were used in our experiments (Additional file 1: Figure S1C, S1D).

### Sphere formation assay

After obtaining the ECSCs in the above manner, cells were cultured in DMEM/F12 (1:1) serum-free medium containing 2% B27 additive, 20 ng/mL EGF, 20 ng/mL bFGF, and 1% penicillin–streptomycin After culture for 3–5 days, 5000 cells were seeded in ultra-low-attachment 6-well plates (Corning, USA) and cultured for 7 days further. Cells were imaged under an inverted fluorescence microscope and an image acquisition system. The sphere diameter was measured at the time of inoculation and after 7 days, respectively.

### CCK-8 assay

Cells were cultured in 96-well plates (Guangzhou Jet Bio-Filtration Co., Ltd.), CCK-8 reagent (10 µL) (Dojindo, Japan) was added to each well, and then incubated at 37 °C with 5% CO_2_ for 3 h. The OD450 value of each well was determined using a microplate reader. Detection was performed at 12 h and 72 h after treatment.

### Transwell invasion assay

Transwell filter inserts (8 μm pore size; Corning) were pre-coated with Matrigel at 37 °C for 30 min. Complete medium (500 µL) was added to each well of the 24-well plate, and pure medium cell suspension (200 µL) was added to the chamber (2 × 10^4^ cells/well). After incubation for 24 h, cells were fixed with 4% poly-oxymethylene for 30 min, then stained with 0.1% crystal violet for 30 min. Cells were imaged under an inverted fluorescence microscope and an image acquisition system (Nikon, Japan).

### Cell cycle analysis

After cell transfection, 1 × 10^6^ cells were collected from each group. After washing once in PBS, cells were fixed in 70% ethanol at 4 °C overnight. Then, cells were washed once in PBS and resuspended in 500 µL PBS, followed by the addition of 10 µL RNase A and 5 µL propidium iodide, then incubated at 37 °C for 30 min. Flow cytometry (BD FACSCalibur, New Jersey, USA) was used to evaluate the proportion of cells at different stages of the cell cycle.

### Apoptosis assay

After cell transfection, 1 × 10^6^ cells were collected from each group. After washing once in PBS, cells were stained with PE Annexin V and 7AAD using PE Annexin V Apoptosis Detection kit (BD Pharmingen™, New Jersey, USA) at room temperature for 15 min. We used flow cytometry (BD FACSCalibur, New Jersey, USA) to evaluate the proportion of apoptotic cells.

### Fluorescence in situ hybridization (FISH)

DNA oligo probes (GenePharma) labeled with FAM for MONC (5′FAM-CAATCAGTTCCTCACATATTAGTCTAAGTCCTCTTTATAATTGAAGAAACATTCTTGTACTGGCTTTATGGGCATTCTTTCAATACTTTCAATGTTAAGAGGCCTTATT-3′) and SA-Biotin for miR-636 (5′Biotin-TGCGGGCGGGACGAGCAAGCACA-3′) were used in the FISH assays, wherein the nuclei were counterstained with 4,6-diamidino-2-phenylindole. All procedures were performed in accordance with the manufacturer’s instructions (GenePharma), and all images were acquired using a confocal laser-scanning microscope (CI: Nikon, Tokyo, Japan).

### Luciferase assay

The bioinformatics website RNAhybrid was used to predict the potential binding sites between MONC and miR-636. MONC wild-type and mutant dual-luciferase reporter vectors were purchased from Liaoning Baihaobio Biotech Co., Ltd. (Liaoning, China), and co-transfected with miR-636 agomir or NC (GenePharma, Shanghai). The bioinformatics website, miRDB, was used to predict the potential binding sites between miR-636 and GLCE. GLCE wild-type and mutant dual-luciferase reporter vectors were purchased from Liaoning Baihaobio Biotech Co., Ltd., and co-transfected with miR-636 agomir or NC. A dual-luciferase reporter gene detection system (Promega, Madison, WI, USA) was used to detect luciferase activity.

### Tumor xenografts in nude mice

Transfected cells were evaluated in vivo in nude mice. We conducted experiments in strict accordance with a protocol approved by the Administrative Panel on Laboratory Animal Care of the Shengjing Hospital (2018PS136K). Four-week-old BALB/C athymic nude mice were purchased from HFK Bioscience (Beijing, China). Each mouse was injected with 5 × 10^6^ cells in the armpit. The tumor volume was calculated using the following formula: tumor volume (mm^3^) = length × width^2^/2. The experiment was conducted in compliance with the Institutional Animal Care and Use Committee standards. When the mice developed tumor metastasis, lethargy, weight loss ≥ 20%, or other signs of discomfort that met the IACUC criteria, the mice were sacrificed by cervical dislocation.

### Statistical analysis

Data are expressed as mean ± standard error of mean (SEM). All statistical analyses were performed using GraphPad Prism 8.0 Software (La Jolla, CA, USA) and SPSS version 22.0 software (Abbott Laboratories, Chicago, IL, USA) through two-sided Student's *t*-test or one-way analysis of variance (ANOVA). Differences were considered statistically significant at *P* < 0.05.

## Results

### MONC exhibits low expression in EC as a tumor suppressor gene

**Fig. 1:**
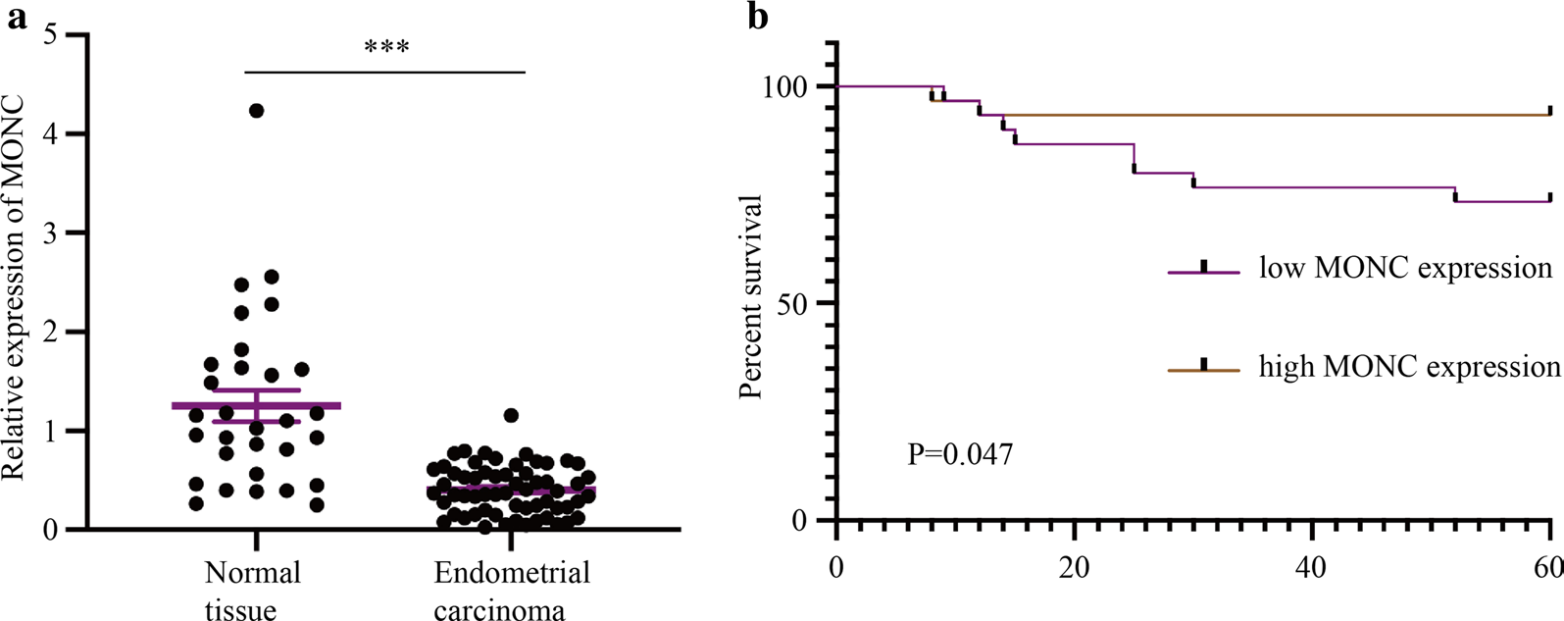
MONC is low expression in EC. **a** qRT-PCR showed the expression of MONC in normal endometrial tissue (n = 30) and human endometrial carcinoma tissue (n = 60). The data are expressed as mean ± SEM. **b** Effect of MONC expression level on EC patient survival. * *P* < 0.05, ** *P* < 0.01, and *** *P* < 0.001

qRT-PCR results revealed that compared with normal human endometrial tissue, MONC exhibited low expression in human EC tissue (Fig. 1a). Clinical pathological analysis showed that MONC expression was related to the invasion depth and FIGO stage (Table 3). The results of survival analysis showed that patients in the MONC high-expression group had a better prognosis (Fig. 1b).

**Table 3:** Relationship between MONC expression and pathologic tumor parameters

Clinical parameters	n	The expression of MONC (Mean ± SEM)	P
Differentiation			0.458
High	28	0.368 ± 0.039	
High-Middle + Middle	25	0.452 ± 0.057	
Middle-Low + Low	7	0.384 ± 0.091	
Invasion depth			0.002 *
< 1/2	40	0.473 ± 0.041	
≥ 1/2	20	0.268 ± 0.035	
FIGO Stage			0.019 *
I	52	0.438 ± 0.032	
II	3	0.105 ± 0.025	
III + IV	5	0.238 ± 0.141	
Age, y			0.241
< 60	34	0.438 ± 0.046	
≥ 60	26	0.362 ± 0.042	

**Fig. 2:**
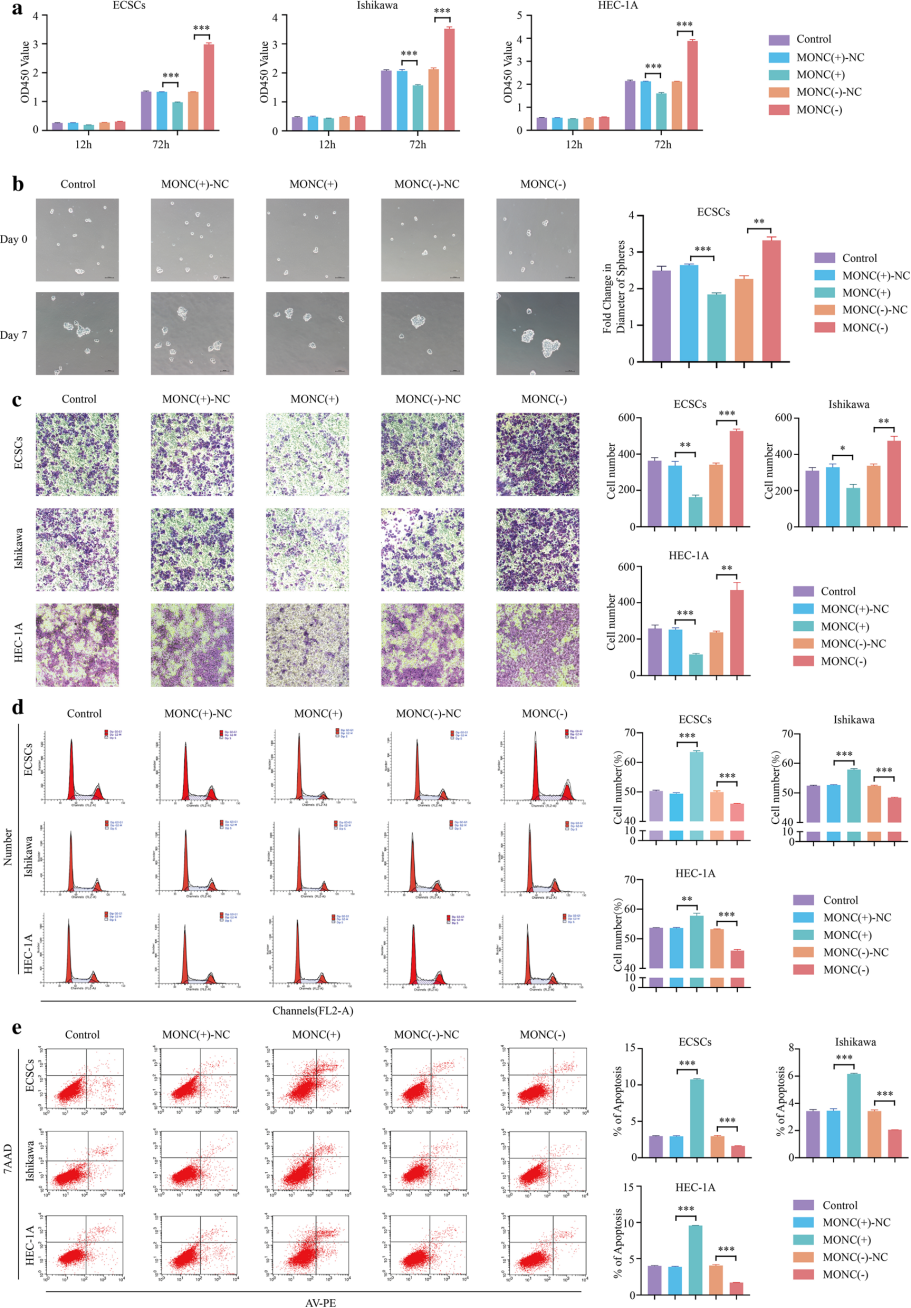
MONC inhibits the malignant biological behavior of ECSCs and ECCs. **a** CCK-8 was used to determine the effect of MONC on the proliferation of ECSCs, and Ishikawa and HEC-1A cell lines. **b** The sphere formation assay was used to measure the effect of MONC on stem cell sphere formation. **c** The Transwell invasion assay was used to determine the effect of MONC on the invasion ability of ECSCs, and Ishikawa and HEC-1A cell lines. **d** Cell cycle analysis was used to detect the effect of MONC on the cell cycle of ECSCs, and Ishikawa and HEC-1A cell lines. **e** Cell apoptosis assay was used to analyze the effect of MONC on apoptosis of ECSCs, and Ishikawa and HEC-1A cell lines. The data are expressed as mean ± SEM (n = 3, each group). * *P* < 0.05, ** *P* < 0.01, and *** *P* < 0.001. (LV-MONC, LV-MONC-RNAi and the corresponding negative control are represented by MONC( +), MONC( +)-NC, MONC(−), MONC(−)-NC in the Figure.)

In the CCK-8 cell proliferation assay, we observed reduced proliferation rates in the MONC overexpression group, while the MONC knocked down group appeared increased (Fig. 2a). The ECSC sphere formation assay showed that the growth rate of ECSC spheroids in the MONC overexpression group slowed down, while the growth rate of ECSC spheroids in the MONC knockdown group accelerated (Fig. 2b). Moreover, the Transwell cell invasion assay revealed that overexpression of MONC inhibited the invasion of ECSCs, Ishikawa cells and HEC-1A cells. Conversely, knockdown of MONC promoted the invasion of ECSCs, and Ishikawa and HEC-1A cells (Fig. 2c). Next, we examined the effects on overexpression and knockdown of MONC on the cell cycle and apoptosis of ECSCs, and Ishikawa and HEC-1A cells using flow cytometry. The results showed that MONC overexpression induced cell cycle arrest in the G0/G1 phase, while knockdown of MONC exerted the opposite effect (Fig. 2d). Overexpression of MONC promoted apoptosis, and knockdown of MONC inhibited apoptosis (Fig. 2e).

Thus, these results indicate that MONC exhibits low expression in EC as a tumor suppressor gene.

### Mir-636 exhibits high expression in EC as an oncogene, while MONC binds and negatively regulates miR-636

**Fig. 3:**
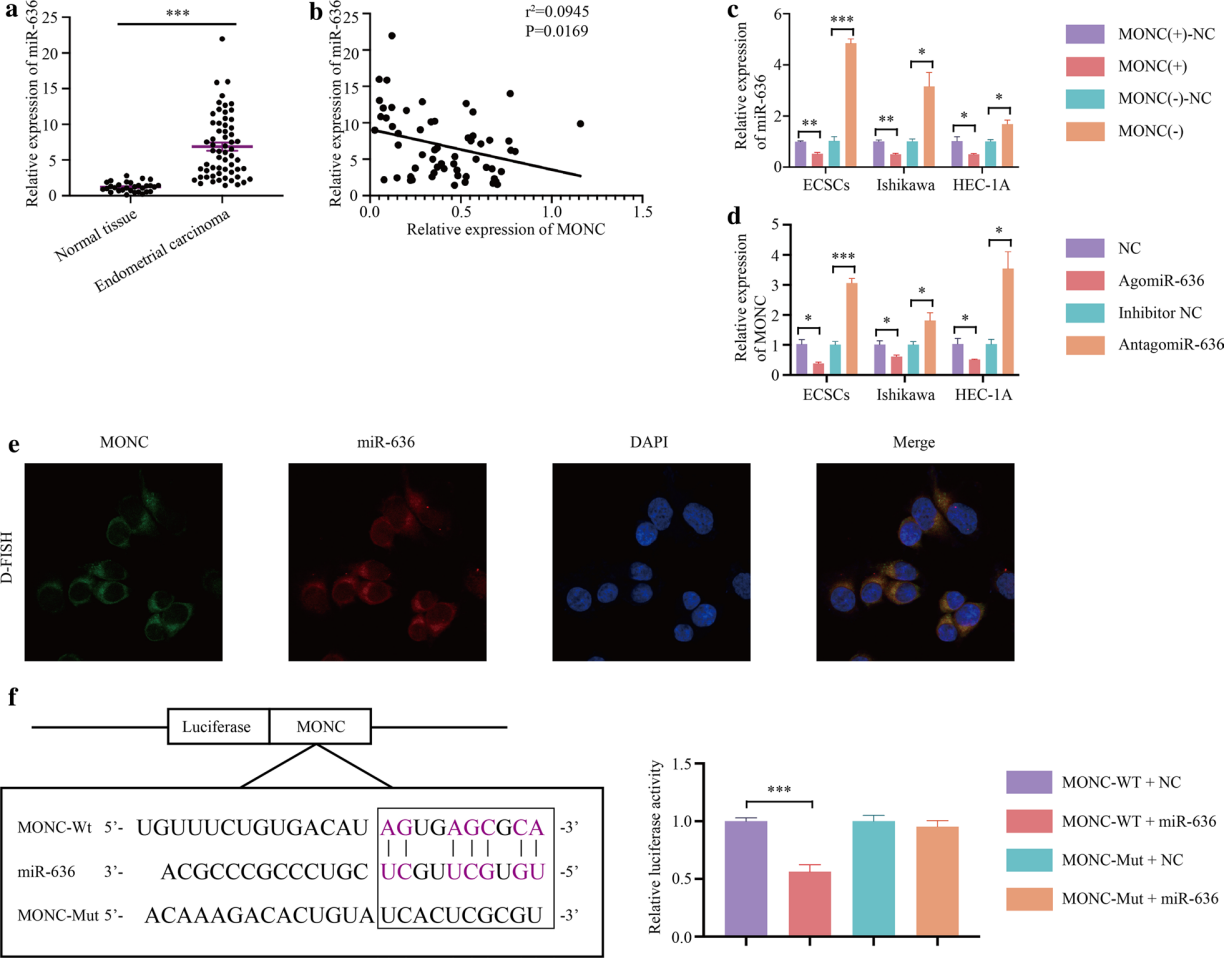
miR-636 is high expression in EC, and MONC is the target of miR-636. **a** qRT-PCR showed the expression of miR-636 in normal endometrial tissue (n = 30) and human endometrial carcinoma tissue (n = 60). **b** Pearson’s test analyzes the correlation between MONC and miR-636. **c** qRT-PCR was used to detect the relative expression of miR-636 after overexpression and knockdown of MONC. **d** qRT-PCR was used to detect the relative expression of MONC after overexpression and knockdown of miR-636. E. MONC and miR-636 were colocalized in Ishikawa cell by FISH. MONC was stained green, miR-636 was stained red, and nuclei were stained blue (DAPI). F. MONC (MONC-WT) predicted miR-636 binding site and the designed mutant sequence (MONC-Mut). Cells were transfected with MONC-WT or MONC-Mut and the indicated miR-636, and subjected to dual luciferase reporter gene analysis. The data are expressed as mean ± SEM (n = 3, each group). * *P* < 0.05, ** *P* < 0.01, and *** *P* < 0.001

qRT-PCR revealed that miR-636 was highly expressed in human EC tissue compared with normal human endometrial tissue (Fig. 3a). Clinical pathological analysis showed that the expression of MONC is related to invasion depth and FIGO stage (Table 4). In addition, the expression of miR-636 was negatively correlated with the expression of MONC (Fig. 3b, Pearson’s rank correlation method: *r*^2^ = 0.0945, *P* = 0.0169).

**Table 4:** Relationship between miR-636 expression and pathologic tumor parameters

Clinical parameters	n	The expression of miR-636 (Mean ± SEM)	P
Differentiation			0.528
High +	28	6.170 ± 0.826	
High-Middle + Middle	25	7.529 ± 0.926	
Middle-Low + Low	7	7.306 ± 1.628	
Invasion depth			0.037 *
< 1/2	40	6.027 ± 0.601	
≥ 1/2	20	8.552 ± 1.167	
FIGO Stage			< 0.001 *
I	52	6.039 ± 0.526	
II	3	10.420 ± 1.008	
III + IV	5	13.37 ± 2.659	
Age, y			0.987
< 60	34	6.877 ± 0.818	
≥ 60	26	6.858 ± 0.800	

We used qRT-PCR to determine the expression of miR-636 in ECSCs, Ishikawa cells, and HEC-1A cells stably transfected with MONC overexpression and knockdown. The results showed that the expression of miR-636 decreased in the MONC overexpression group, while miR-636 expression increased in the MONC knockdown group (Fig. 3c). Next, we transfected ECSCs, Ishikawa cells, and HEC-1A cells with miR-636 agomir, miR-636 antagomir, and corresponding negative controls. Using qRT-PCR, we found that MONC expression was decreased in the miR-636 agomir group and increased in the miR-636 antagomir group (Fig. 3d).

The double FISH (D-FISH) assay showed that MONC and miR-636 were relatively co-localized in the cytoplasm of Ishikawa cells (Fig. 3e). A bioinformatics database (RNAhybrid) was used to predict the binding site between MONC and miR-636. In order to further study whether MONC is a functional target of miR-636, we co-transfected HEK-293 T cells with MONC-WT and NC, and MONC-WT and miR-636. Thereafter, we performed a dual-luciferase reporter assay. The results showed that the relative luciferase activity of the MONC-WT + miR-636 group was lower than that of the MONC-WT + NC group, suggesting that there is a binding site between MONC and miR-636 (Fig. 3f). A MONC mutant vector was constructed, based on the predicted binding sites of the bioinformatics database (RNAhybrid). Next, we co-transfected HEK-293 T cells with MONC-Mut and NC, and MONC-Mut and miR-636. Detection of the dual-luciferase reporter assay verified the binding site between MONC and miR-636 (Fig. 3f).

Therefore, we speculate that miR-636 is overexpressed in EC as an oncogene, MONC binds and negatively regulates miR-636, and there may be a feedback loop of mutual inhibition between MONC and miR-636.

### Knockdown of miR-636 mediates the tumor suppressive effect of MONC overexpression in ECSCs and ECCs

To determine whether miR-636 mediated tumor suppression via MONC overexpression, we first transfected miR-636 antagomir into ECSCs, Ishikawa cells, and HEC-1A cells stably overexpressing MONC. miR-636 agomir was transfected into MONC knockdown ECSCs, Ishikawa cells, and HEC-1A cells. We divided the experiments into six groups: Control, MONC ( +), miR-636 (−), Stable NC, MONC( +) + miR-636(−), and MONC(-) + miR-636( +).

**Fig. 4:**
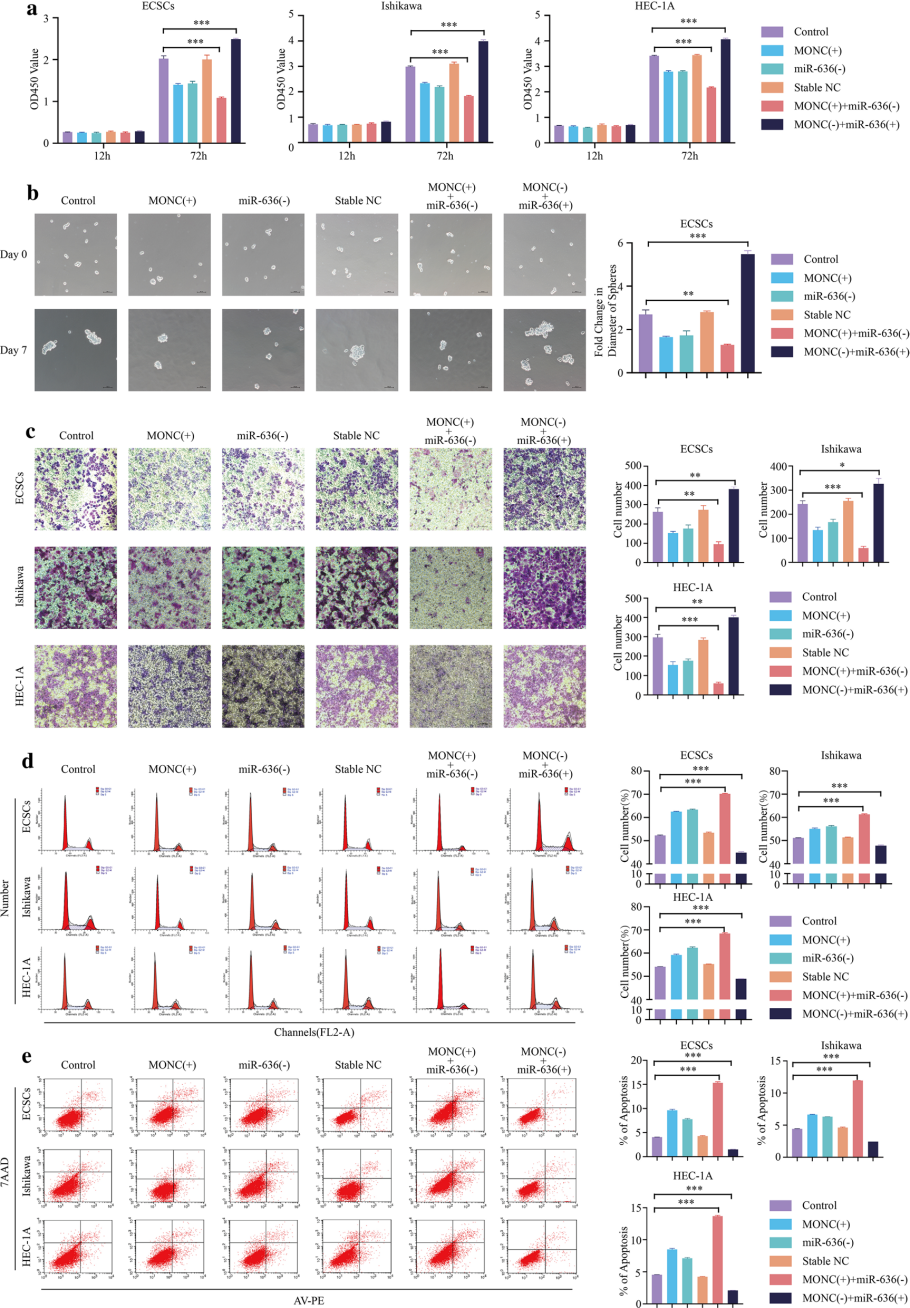
Knockdown of miR-636 mediates tumor suppressive effects of MONC overexpression in ECSCs, and ECCs. **a** CCK-8 was used to determine the effects of MONC and miR-636 on the proliferation of ECSCs, and Ishikawa and HEC-1A cell lines. **b** The sphere formation assay was used to determine the effect of MONC and miR-636 on stem cell sphere formation. **c** The Transwell invasion assay was used to determine the effect of MONC and miR-636 on the invasion ability of ECSCs, and Ishikawa and HEC-1A cell lines. **d** Cell cycle analysis was used to determine the effects of MONC and miR-636 on the cell cycle of ECSCs, and Ishikawa and HEC-1A cell lines. **e** Cell apoptosis assay was used to analyze the effects of MONC and miR-636 on the apoptosis of ECSCs, and Ishikawa and HEC-1A cell lines. The data are expressed as mean ± SEM (n = 3, each group). * P < 0.05, ** *P* < 0.01, and *** *P* < 0.001. (LV-MONC, LV-MONC-RNAi, agomiR-636 and antagomir-636 are represented by MONC( +), MONC(−), miR-636( +) and miR-636(−) in the Figure.)

The CCK-8 cell proliferation assay indicated that the MONC( +) + miR-636(-) group exhibited the lowest proliferation rate, while the MONC(−) + miR-636( +) group exhibited the highest proliferation rate (Fig. 4a). The sphere formation assay demonstrated that miR-636 knockdown inhibited the growth rate of ECSC spheroids. The MONC( +) + miR-636(-) group displayed the most obvious inhibitory effect on the growth rate of ECSC spheroids. The ECSC spheroids in the MONC(−) + miR-636( +) group displayed the fastest growth rate (Fig. 4B). According to the Transwell cell invasion experiment, we found that knockdown of miR-636 inhibited the invasion of ECSCs, Ishikawa cells, and HEC-1A cells. The MONC( +) + miR-636(−) group displayed the strongest invasion inhibitory effects in ECSCs, Ishikawa cells, and HEC-1A cells, while the MONC(−) + miR-636( +) group displayed the weakest invasion inhibitory effects (Fig. 4c). Thereafter, we employed flow cytometry to observe the cell cycle and apoptosis of ECSCs, Ishikawa cells, and HEC-1A cells after transfection. The results showed that miR-636 antagomir induced cell cycle arrest at the G0/G1 phase, the MONC ( +) + miR-636(−) group had the strongest ability to induce cell cycle arrest in the G0/G1 phase, and the MONC(−) + miR-636( +) group had the weakest ability (Fig. 4D). miR-636 inhibited apoptosis, and the MONC( +) + miR-636(−) group exhibited the strongest inhibitory effect on apoptosis, while the MONC(−) + miR-636( +) group exhibited the weakest (Fig. 4e).

Therefore, we believe that miR-636 can mediate tumor suppressive effects of MONC overexpression in ECSCs, Ishikawa cells, and HEC-1A cells, and that knockdown of MONC combined with overexpression of miR-636 have significant carcinoma-promoting effects in ECSCs and ECCs.

### GLCE exhibits low expression in EC as a tumor suppressor gene, and participates in the malignant progression of ECSCs induced by MONC-miR-636

**Fig. 5:**
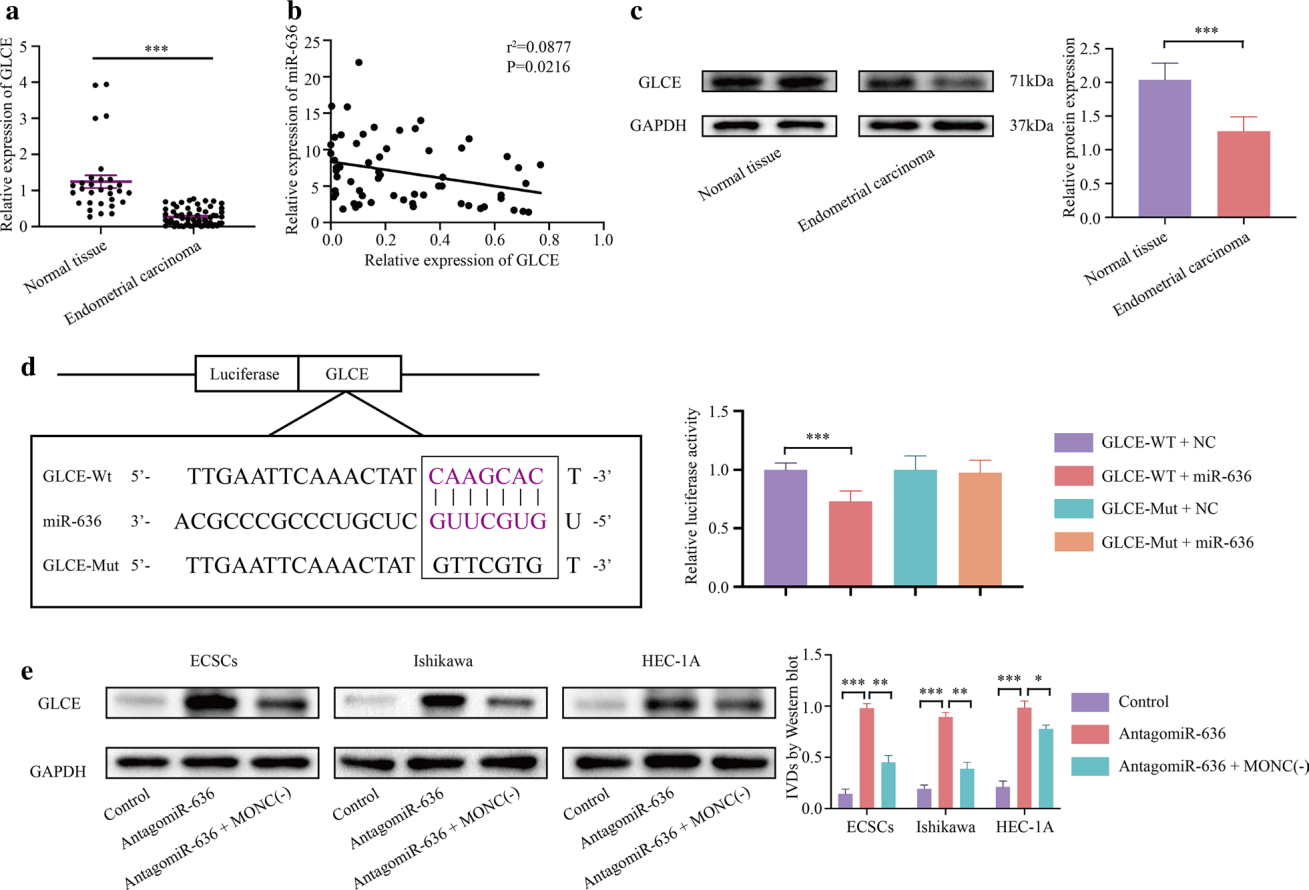
GLCE is low expression in EC and participates in the MONC-miR-636 regulatory axis. **a** qRT-PCR showed the expression of *GLCE* in normal endometrial tissue (n = 30) and human endometrial carcinoma (n = 60). **b** Pearson’s test analyzes the correlation between GLCE and miR-636. **c** Western blot showed the expression in GLCE normal endometrial tissue (n = 30) and human endometrial carcinoma (n = 60). **d** GLCE (GLCE-WT) predicted miR-636 binding site and the designed mutant sequence (GLCE-Mut). Cells were transfected with GLCE-WT or GLCE-Mut and the indicated miR-636, and then subjected to dual luciferase reporter gene analysis. **e** Western blot was used to observe the effects of MONC and miR-636 on GLCE expression in ECSCs, and Ishikawa and HEC-1A cell lines. The data are expressed as mean ± SEM (n = 3, each group). * *P* < 0.05, ** *P* < 0.01, and *** *P* < 0.001

qRT-PCR and western blot results revealed low expression of GLCE in human EC tissue compared to normal human endometrial tissue (Fig. 5a, c). Clinical pathological analysis showed that the expression of GLCE is related to invasion depth and FIGO stage (Table 5). In addition, the expression of miR-636 was negatively correlated with the expression of GLCE (Fig. 5b, Pearson’s rank correlation method: *r*^2^ = 0.0877, *P* = 0.0216).

**Table 5:** Relationship between GLCE expression and pathologic tumor parameters

Clinical parameters	n	The expression of GLCE (Mean ± SEM)	P
Differentiation			0.096
High	28	0.197 ± 0.039	
High-Middle + Middle	25	0.323 ± 0.050	
Middle-Low + Low	7	0.349 ± 0.090	
Invasion depth			0.023 *
< 1/2	40	0.316 ± 0.039	
≥ 1/2	20	0.170 ± 0.042	
FIGO Stage			0.038 *
I	52	0.297 ± 0.033	
II	3	0.017 ± 0.011	
III + IV	5	0.110 ± 0.019	
Age, y			0.104
< 60	34	0.311 ± 0.042	
≥ 60	26	0.210 ± 0.043	

Using a bioinformatics database (miRDB), we identified GLCE as a downstream target gene of miR-636. Therefore, we co-transfected HEK-293 T cells with GLCE-WT and NC, and GLCE-WT and miR-636. Dual-luciferase reporter assay revealed that the relative luciferase activity of the GLCE-WT + miR-636 group was lower than that of the GLCE-WT + NC group, suggesting the presence of a binding site between GLCE and miR-636 (Fig. 5d). A GLCE mutant vector was constructed based on the predicted binding sites from the bioinformatics database (miRDB), followed by co-transfection of GLCE-Mut and NC, and GLCE-Mut and miR-636. The dual-luciferase reporter test verified the binding site between MONC and miR-636 (Fig. 5d).

In order to investigate whether MONC regulates miR-636 in EC as a competitive endogenous RNA (ceRNA) of GLCE, western blot was used to determine GLCE expression level in ECSCs, Ishikawa cells, and HEC-1A cells transfected with MONC. Transfection with miR-636 antagomir promoted GLCE expression at the protein level. Simultaneously, MONC knockdown rescued transfection with miR-636 antagomir from promoting GLCE protein expression (Fig. 5e).

**Fig. 6:**
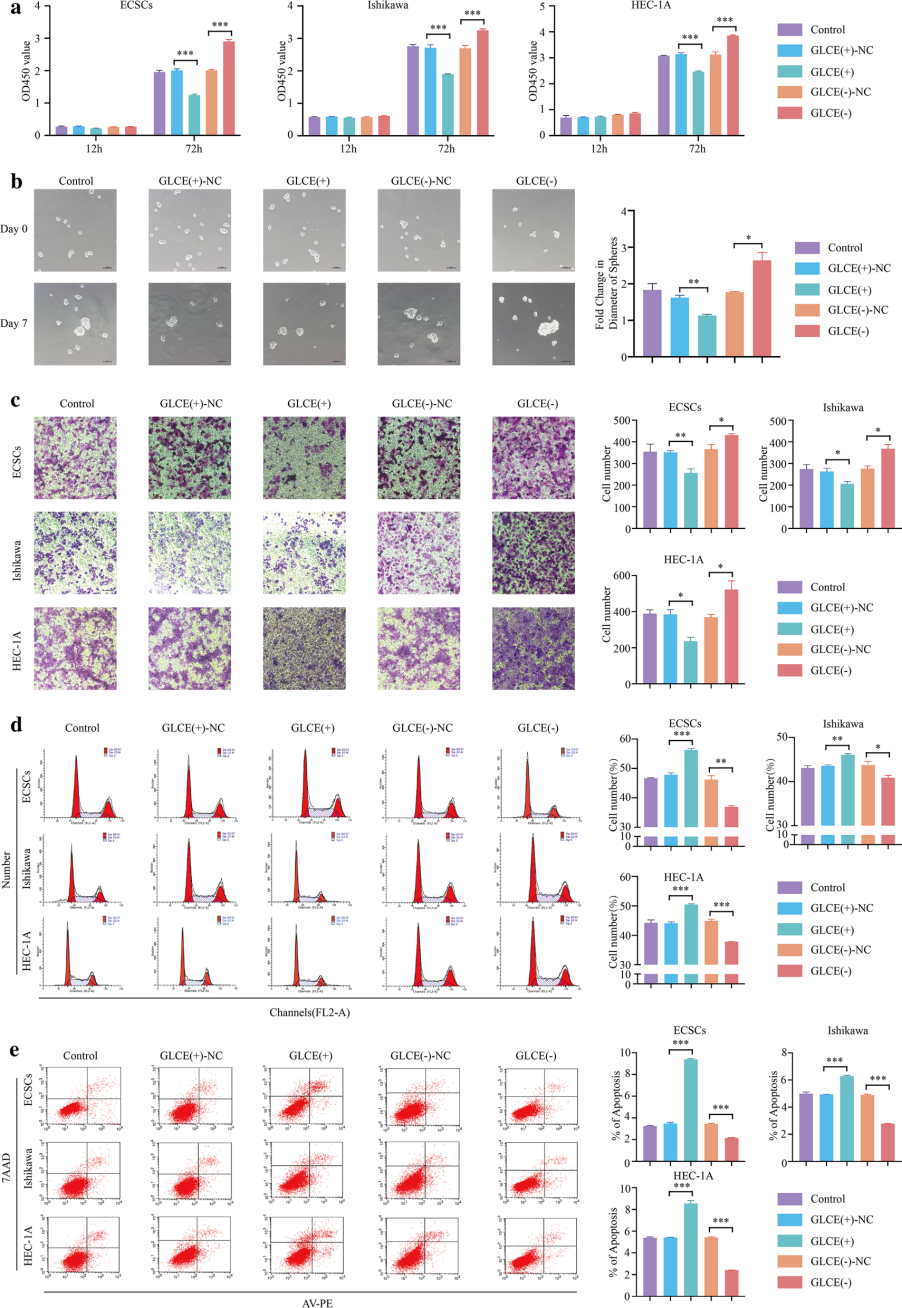
GLCE inhibits the malignant biological behavior of ECSCs and ECCs. **a** CCK-8 was used to observe the effect of GLCE on the proliferation of ECSCs, and Ishikawa and HEC-1A cell lines. **b** The sphere formation assay was used to determine the effect of GLCE on stem cell sphere formation. **c** The Transwell invasion assay was used to determine the effect of GLCE on the invasion ability of ECSCs, and Ishikawa and HEC-1A cell lines. **d** Cell cycle analysis was used to detect the effect of GLCE on the cell cycle of ECSCs, and Ishikawa and HEC-1A cell lines. **e** Cell apoptosis assay was used to analyze the effect of GLCE on apoptosis of ECSCs, and Ishikawa and HEC-1A cell lines. The data are expressed as mean ± SEM (n = 3, each group). * *P* < 0.05, ** *P* < 0.01, and *** *P* < 0.001. (GLCE, GLCE-RNAi and the corresponding negative control are represented by GLCE( +), GLCE( +)-NC, GLCE(−), GLCE(−)-NC in the Figure.)

To explore the effect of GLCE on the malignant biological behavior of ECSCs, we first performed a CCK-8 cell proliferation assay and found that the proliferation rate in the GLCE-overexpressed group decreased, while that in the GLCE-knockdown group increased (Fig. 6a). The sphere formation assay indicated that the growth rate of ECSC spheroids in the GLCE overexpression group slowed down, while that in the GLCE knockdown group accelerated (Fig. 6b). We observed that the overexpression of GLCE inhibited the invasion of ECSCs, Ishikawa cells, and HEC-1A cells. Conversely, knockdown of GLCE promoted the invasion of ECSCs, Ishikawa cells, and HEC-1A cells (Fig. 6c). Next, we used flow cytometry to examine the effects of GLCE knockdown and overexpression on the cell cycle and apoptosis of ECSCs, Ishikawa cells, and HEC-1A cells. The results showed that overexpression of GLCE induced cell cycle arrest at the G0/G1 phase, while knockdown of GLCE exerted the opposite effect (Fig. 6d). Overexpression of GLCE promoted apoptosis, and GLCE knockdown inhibited apoptosis (Fig. 6e).

Therefore, we speculated that GLCE expression is low in EC as a tumor suppressor gene and that GLCE participates in the malignant progression of ECSCs and ECCs induced by MONC-miR-636. In these cells, MONC can regulate miR-636 as the ceRNA of GLCE.

### Overexpression of MONC inhibits the EMT process in ECSCs and ECCs, while miR-636 rescues this inhibitory effect

**Fig. 7:**
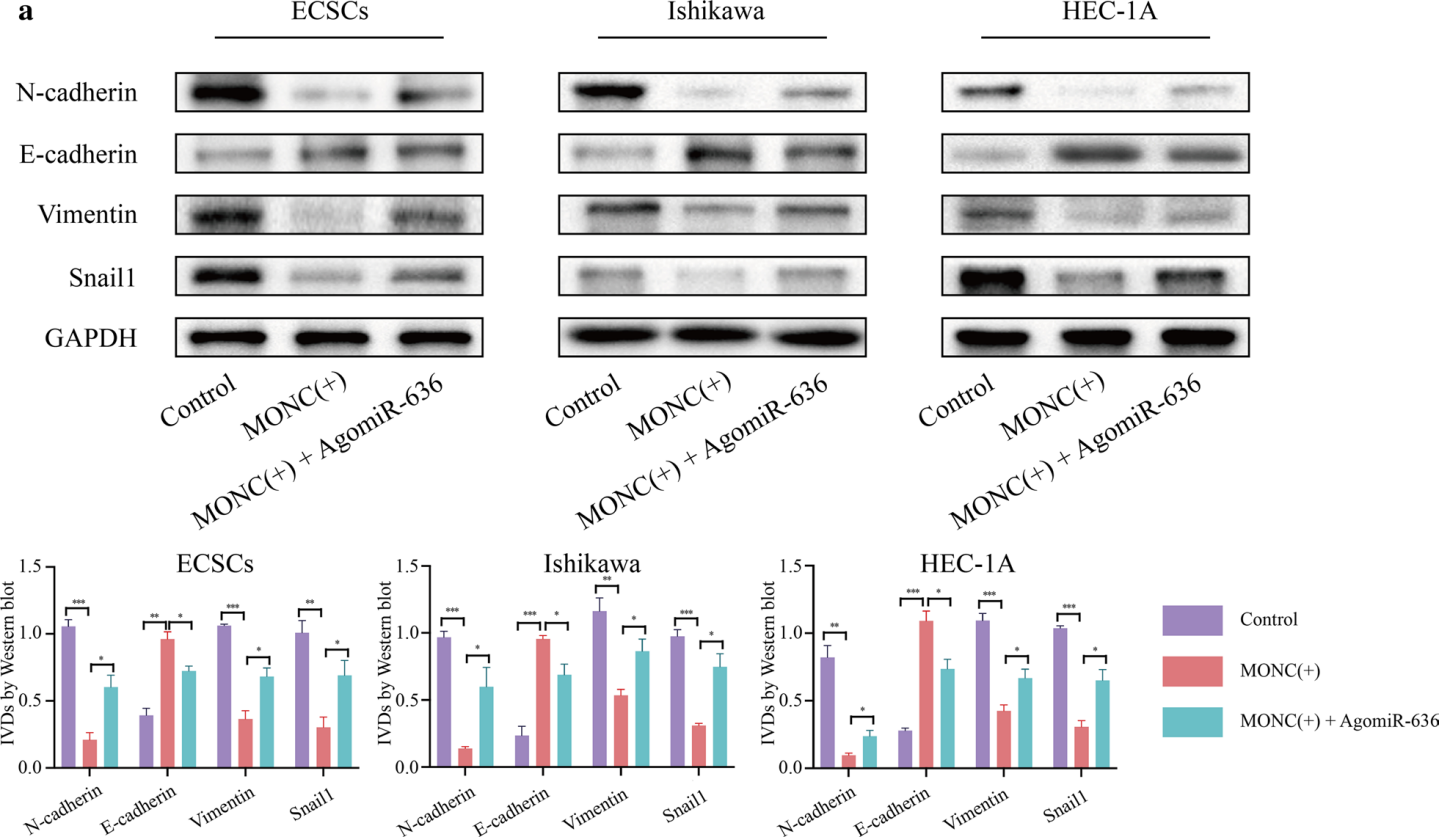
miR-636 rescued the inhibitory effect of MONC on EMT process in ECSCs, and ECCs.** a** Western blot was used to observe the effects of MONC and miR-636 on EMT process in ECSCs, and Ishikawa and HEC-1A cell lines. The data are expressed as mean ± SEM (n = 3, each group). * *P* < 0.05, ** *P* < 0.01, and *** *P* < 0.001. (LV-MONC and agomiR-636 are represented by MONC( +) and miR-636( +) in the Figure.)

In order to explore the molecular mechanism of the downstream signaling pathway regulated by MONC and miR-636, we first constructed ECSCs, Ishikawa cells and HEC-1A cell lines stably overexpressing MONC. miR-636 agomir was transfected into ECSCs, Ishikawa cells, and HEC-1A cells stably overexpressing MONC. We divided the experiment into three groups, namely, Control, MONC( +), and MONC( +) + miR-636( +). The protein levels of EMT-related indicators were detected using western blot. The results showed that MONC overexpression inhibited the expression of Snail1, Vimentin, and N-cadherin, and promoted the expression of E-cadherin. miR-636 rescued the inhibitory effects of MONC overexpression on EMT process (Fig. 7a).

Therefore, we speculate that the knockdown of MONC in combination with miR-636 inhibits EMT process in ECSCs and ECCs.

### Overexpression of MONC inhibited the growth of EC tumors in nude mice and miR-636 rescued this inhibitory effect

**Fig. 8:**
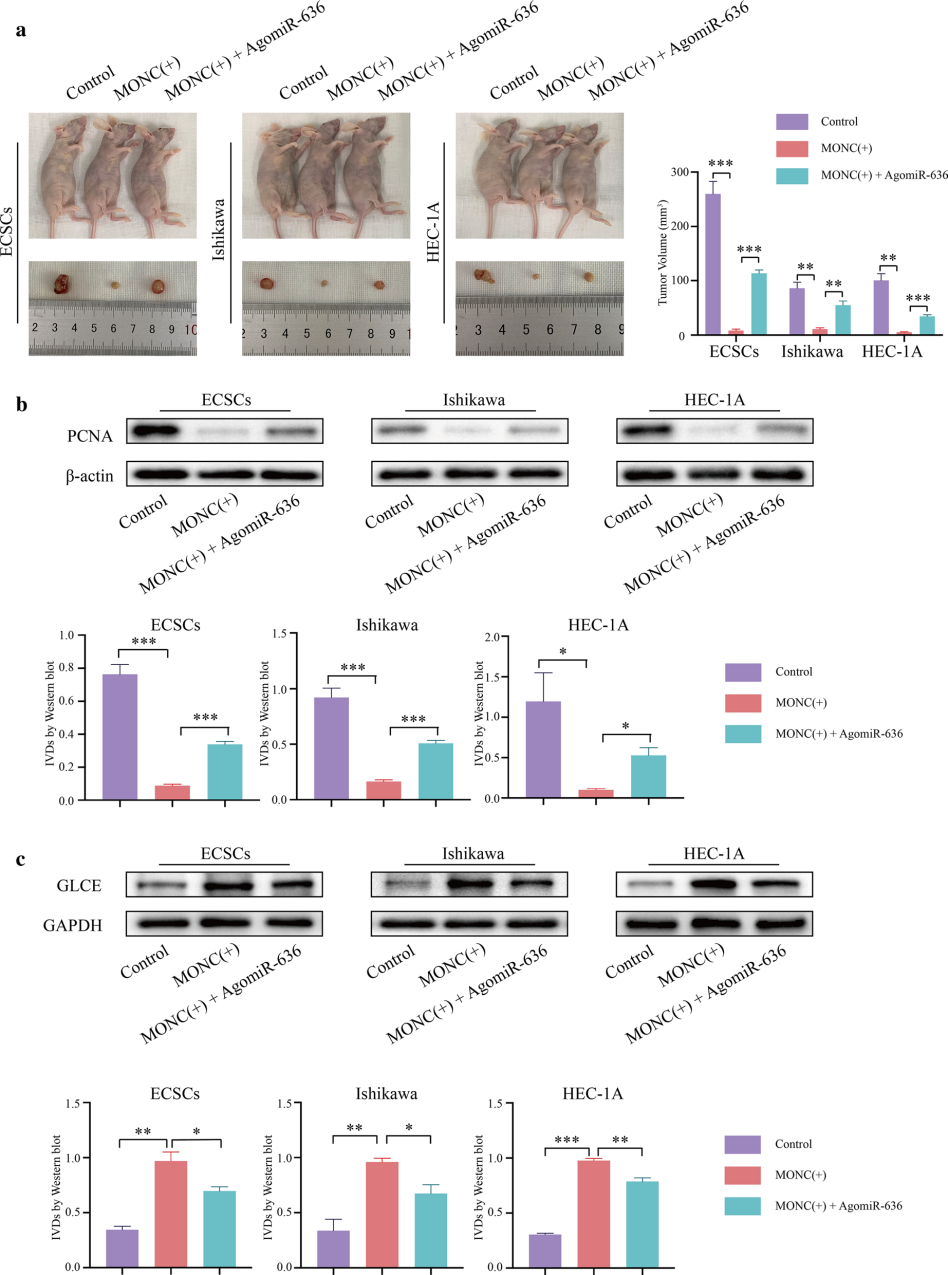
In vivo study of tumor xenografts. **a** The tumor at the end point forms an image. **b** Western blot was used to detect the expression of PCNA in Control group, MONC overexpression group, and MONC overexpression co-transfected with agomiR-636 group. **c** Western blot was used to detect the expression of GLCE in Control group, MONC overexpression group, and MONC overexpression co-transfected with agomiR-636 group. The data are expressed as mean ± SEM (n = 3, each group). * *P* < 0.05, ** *P* < 0.01, and *** *P* < 0.001. (LV-MONC and agomiR-636 are represented by MONC( +) and miR-636( +) in the Figure.)

We conducted nude mice tumorigenesis experiments to investigate the effect of MONC combined with miR-636 on tumor growth in nude mice. MONC( +) and MONC( +) + miR-636( +) ECSCs, Ishikawa cells, and HEC-1A cells were injected subcutaneously into nude mice. The results are shown in Fig. 8. In MONC( +) and the MONC( +) + miR-636( +) groups, the tumor volume was smaller than that in the control group, and the MONC( +) group exhibited the smallest tumor volume (Fig. 8a). Further, we detected the expression of PCNA in tumors by western blot. The expression of PCNA in MONC( +) and MONC( +) + miR-636( +) groups decreased, and was the lowest in the MONC( +) group(Fig. 8b). Then we detected the expression of GLCE in the tumor using western blot. The expression of GLCE in the tumors of MONC( +) and MONC( +) + miR-636( +) groups increased, while was the highest in the MONC( +) group (Fig. 8c). Results indicate that MONC inhibited the growth of EC tumors, and miR-636 rescued MONC from inhibiting the growth of EC tumors.

## Discussion

EC is one of the three major malignant tumors of the female reproductive system [1]. The risk factors are related to age, obesity, hypertension, excessive endometrial exposure to estrogen, diabetes, and hereditary nonpolyposis colorectal cancer. At present, the main treatment is still total hysterectomy with bilateral salpingo-oophorectomy, while radiation and chemotherapy can also play therapeutic roles [18].

CSCs can promote tumor growth, recurrence, metastasis, and drug resistance [19]. The functional role of CSCs in the generation and recurrence of malignant tumors has two key characteristics: the self-renewal capacity of CSCs and the potential to differentiate into an infinite group of heterogeneous cancer cells [20]. Owing to the plasticity of CSCs, stationary CSCs may produce cycling CSCs, leading to cancer recurrence [21]. Studies have shown that PKA activation promotes CSC state in small cell lung cancer [22]. In colorectal cancer, CSCs evade treatment-mediated DNA damage by changing cell cycle checkpoints, increasing DNA damage repair capabilities, and effectively removing reactive oxygen species [23]. In recent years, there have been reports on ECSCs, but there are few related studies. Nevertheless, studies have found selective and specific effects of metformin on the activity of ECSCs [24]. In another study, SPIONs were found to be highly efficient nanocarriers for nucleic acids. Loading miR-326 on these carriers inhibited the activation of the GPR91/STAT3/VEGF signaling pathway and significantly reduced ECSC activity [25]. Moreover, our group used serum-free suspension culture to isolate ECSCs from Ishikawa cells in a previous research [7]. In the present study, we used serum-free suspension culture to isolate ECSCs from Ishikawa cells for subsequent experiments.

The mechanism of action of LncRNA in different tumors is inconsistent, and includes changes in histone modification, regulation of classic stem cell-related signaling pathways (such as SOX2/KLF4), induction of EMT, and inhibition of miRNA function [26]. Abnormal expression of LncRNA plays a key role in self-renewal, effective transformation of CSCs, and tumor progression. For example, the well-known LncRNA HOTAIR is significantly up-regulated and promotes CSC properties in breast and colon CSCs [27, 28]. LncRNA HAND2-AS1 promotes self-renewal of liver CSCs and drives liver cancer [29]. LncRNA DLX6-AS1 down-regulation may inhibit methylation of the CADM1 promoter and the inactivation of the STAT3 signaling pathway as well as up-regulate CADM1 to suppress stem cell characteristics of liver CSCs [30].

MONC has been reported to be upregulated in AML [13] and associated with survival in head and neck squamous cell carcinoma patients [10]. In addition, recent study has found that MONC contributes to malignant phenotypes of gastric cancer and may become a promising therapeutic target [31]. In this study, we found low expression of LncRNA MONC in EC. Moreover, patients in the MONC high-expression group had a better prognosis. MONC up-regulation inhibited the growth rate of ECSCs spheres, while inhibiting proliferation and invasion, promoting apoptosis, and inducing cell cycle arrest at the G0/G1 phase in ECSCs, Ishikawa cells and HEC-1A cells.

LncRNA can show similar effects as tumor suppressor genes or oncogenes by affecting various cellular processes related to cancer, including cell growth, metastasis, differentiation, and stemness [32]. LncRNA can act as ceRNA or a natural miRNA sponge, communicate with each other, and regulate together through the combination of competition and shared microRNA [33]. For example, LncRNA HOTTIP facilitates the development of breast cancer by regulating the miR-148a-3p/WNT1 pathway [34]. LncRNA ZEB2-AS1, through the miR-574-3p/HMGA2 axis, promotes the proliferation, migration, and invasion of esophageal squamous cell carcinoma cells [35]. Previous research indicates that PVT1 participates in the ceRNA regulatory network in EC and regulates the expression and function of miR-195-5p as a ceRNA or miRNA sponge [36], and LINC01016 promotes the malignant phenotype of ECCs by regulating the miR-302a-3p/miR-3130-3p/NFYA/SATB1 axis [37]. In related research on EC and ECSCs, Linc-RNA-RoR acts as a "sponge," preventing microRNA-145 from mediating ECSCs differentiation [38].

Using bioinformatics websites, we predicted a binding site between MONC and miR-636 and verified the same by dual-luciferase reporter gene detection. Further investigations to determine the mechanism by which MONC regulates miR-636 to affect ECSCs and ECCs, revealed that MONC and miR-636 were relatively co-localized in the cytoplasm of Ishikawa cells, as determined by D-FISH. In addition, miR-636 knockdown could mediate tumor suppression associated with MONC overexpression in ECSCs and ECCs. This provides an insight into the tumor suppressive effect of MONC and miR-636.

Further explorations into the regulatory mechanism of MONC, revealed low expression of GLCE, a tumor suppressor gene, in EC and that GLCE mRNA is the target of miR-636. MONC up-regulation promoted GLCE protein expression, while miR-636 rescued the promotion effect of MONC up-regulation on GLCE. Functional experiments showed that GLCE up-regulation inhibited the growth rate of ECSC spheres, while also inhibiting proliferation and invasion, promoting apoptosis, and inducing cell cycle arrest at the G0/G1 phase in ECSCs, Ishikawa cells, and HEC-1A cells. We believe that the MONC/miR-636/GLCE axis plays a crucial role in sphere formation, proliferation, invasion, apoptosis, and induction of cell cycle arrest in ECSCs and ECCs. This information could provide future treatment options for EC.

Most tumors are affected by EMT, the process of acquiring mesenchymal features from epithelial cells, during tumor progression. It is generally believed that cancers derived from epithelia are determined by the EMT process [39]. The weakened adhesion between tumor cells and the increased mobility of tumor cells are the basis for tumor invasion and metastasis, and the invasion ability of tumor cells is significantly enhanced after the occurrence of EMT [40]. In human malignant tumors, among the transcription factors involved in EMT, Snail plays a major inducing role, while Twist and Zeb1/2 are mainly involved in retaining the aggressive mesenchymal phenotype. Activation of EMT is related to the generation of CSCs, and a connection exists between EMT, stemness, and the metastatic initiation potential of tumor cells [41]. In this study, MONC overexpression inhibited the expression of Snail1, Vimentin, and N-cadherin, and promotes the expression of E-cadherin. Further, miR-636 rescued the inhibitory effect of MONC overexpression on EMT. Therefore, we speculate that in ECSCs, MONC and combined with miR-636 inhibit tumor EMT. Moreover, our in vivo study indicated that MONC can inhibit the growth of EC tumors, while miR-636 rescued the inhibition of EC tumor growth due to MONC.

Based on these results, we can conclude that MONC overexpression combined with miR-636 knockdown has tumor suppressor function in vivo and in vitro, and there is a negative interaction between these two factors. Therefore, in summary, the MONC/miR-636/GLCE axis may play an important role in human endometrial cancer, and put forward a promising treatment target for endometrial cancer.

## Conclusion

Our study confirmed, for the first time, that MONC inhibits the malignant biological behavior of ECSCs and ECCs by directly inhibiting miR-636. In addition, miR-636 may indirectly reduce the expression of MONC. Down-regulation of miR-636 may promote the expression of GLCE by targeting its 3′-untranslated region (UTR), thereby inhibiting the progression of ECSCs and ECCs. MONC combined with miR-636 inhibited the tumor EMT. We believe that the MONC/miR-636/GLCE axis may provide a new therapeutic strategy for the treatment of human EC.

## Supplementary Information

**Additional file 1: Figure S1**. Screening for MONC and GLCE knockdownand sorting of flow cytometry of Ishikawa cells. A. Lentivirus screening forMONC knockdown. B. Plasmid screening for GLCE knockdown. A and B, dataare expressed as mean ± SEM (n=3, each group). * P <0.05, ** P <0.01, and*** P <0.001. C. Ishikawa cell line sorting by flow cytometry and obtainedECSCs. D. ECSCs under 10 × and 40 × magnification.

## Data Availability

All data generated or analyzed during this study are included in this published article.

## Abbreviations

ECSCs: Endometrial cancer stem cells
ECCs: Endometrial cancer cells
EC: Endometrial carcinoma
UTR: Untranslated region
EMT: Epithelial-to-mesenchymal transition
CSCs: Cancer stem cells
LncRNA: Long non-coding RNA
miRNA: MicroRNA
FBS: Fetal bovine serum
PMSF: Phenylmethanesulfonyl fluoride
ANOVA: One-way analysis of variance
qRT-PCR: Quantitative real-time polymerase chain reaction
ceRNA: Competitive endogenous RNA
AML: Acute megakaryoblastic leukemia
