# Supplementary figures and images for: LncRNA MONC suppresses the malignant phenotype of Endometrial Cancer Stem Cells and Endometrial Carcinoma Cells by regulating the MiR-636/GLCE axis
Source: Cancer Cell Int. 2021 Jun 30;21:331. doi: 10.1186/s12935-021-01911-1 (PMC8243592; doi:10.1186/s12935-021-01911-1)

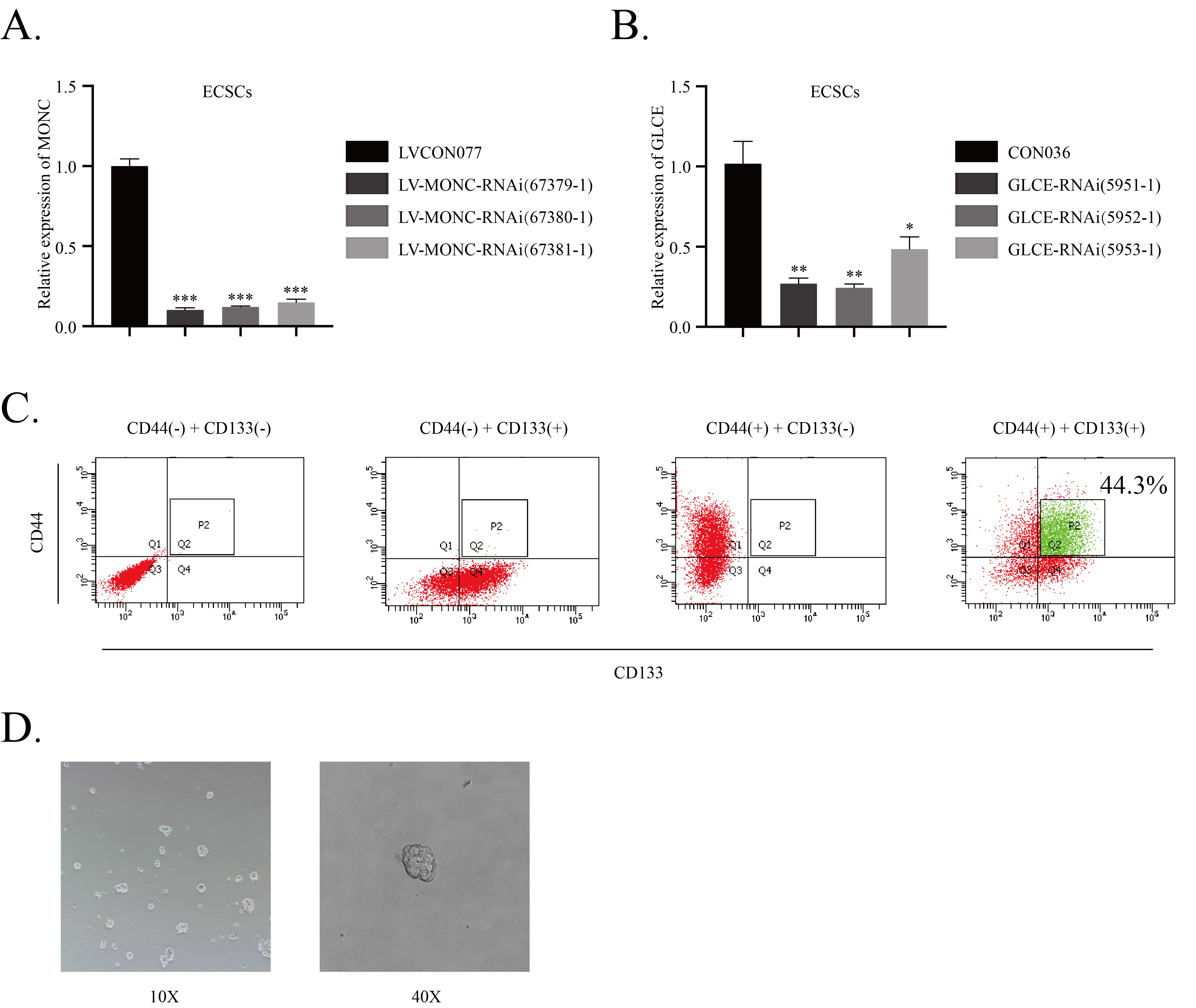

Supplement: Supplementary file 1 — Additional file 1: Figure S1. Screening for MONC and GLCE knockdownand sorting of flow cytometry of Ishikawa cells. A. Lentivirus screening forMONC knockdown. B. Plasmid screening for GLCE knockdown. A and B, dataare expressed as mean ± SEM (n=3, each group). * P <0.05, ** P <0.01, and*** P <0.001. C. Ishikawa cell line sorting by flow cytometry and obtainedECSCs. D. ECSCs under 10 × and 40 × magnification. [file 12935_2021_1911_MOESM1_ESM.jpg]
